# Visualizing and Optimizing Phase Matching in Nonlinear Guided‐Mode Resonators With the Green's Function Integral Method

**DOI:** 10.1002/nap2.70091

**Published:** 2026-04-20

**Authors:** Chengkang Liang, Quanying Li, Jiale Xu, Pingqi Gao, Jiancan Yu

**Affiliations:** ^1^ School of Materials Sun Yat‐Sen University Shenzhen China

**Keywords:** guided‐mode resonance, nonlinear optics, phase analysis, phase‐matching factor, second‐harmonic generation

## Abstract

Efficient nonlinear frequency conversion in nanophotonics requires not only strong fundamental field but also precise phase matching among distributed nonlinear sources. Here, we develop the two‐dimensional Green's function integral method (GFIM) to directly visualize and optimize phase matching in nonlinear guided‐mode resonators. Using GFIM phase analysis, we generalize the phase‐matching factor (PMF) as a rigorous metric of spatial phase coherence in harmonic generation, revealing severe phase mismatch in conventional guide mode resonators. Guided by phase‐matching profiles, we propose design strategies to improve the phase coherence, particularly by introducing a high‐index waveguide layer that confines the fundamental field in the nonlinear material to regions where the harmonic Green's function varies slowly. This configuration achieves a PMF exceeding 0.91, approaching the ideal value of unity, and yields a record SHG efficiency of 26.7% at a low pump intensity of 2 kW/cm^2^. These results establish the microscopic phase‐matching visualization as an effective strategy for compact high‐performance nonlinear photonic devices.

## Graphical abstract

The Green’s function integral framework provides a unified physical picture of microscopic phase mismatch and introduces a phase‐matching factor (PMF) as a quantitative design metric for harmonic coherence. By rational phase engineering, this approach achieves a PMF of 0.91 and an SHG efficiency of 26.7% in compact nonlinear nanophotonic devices.

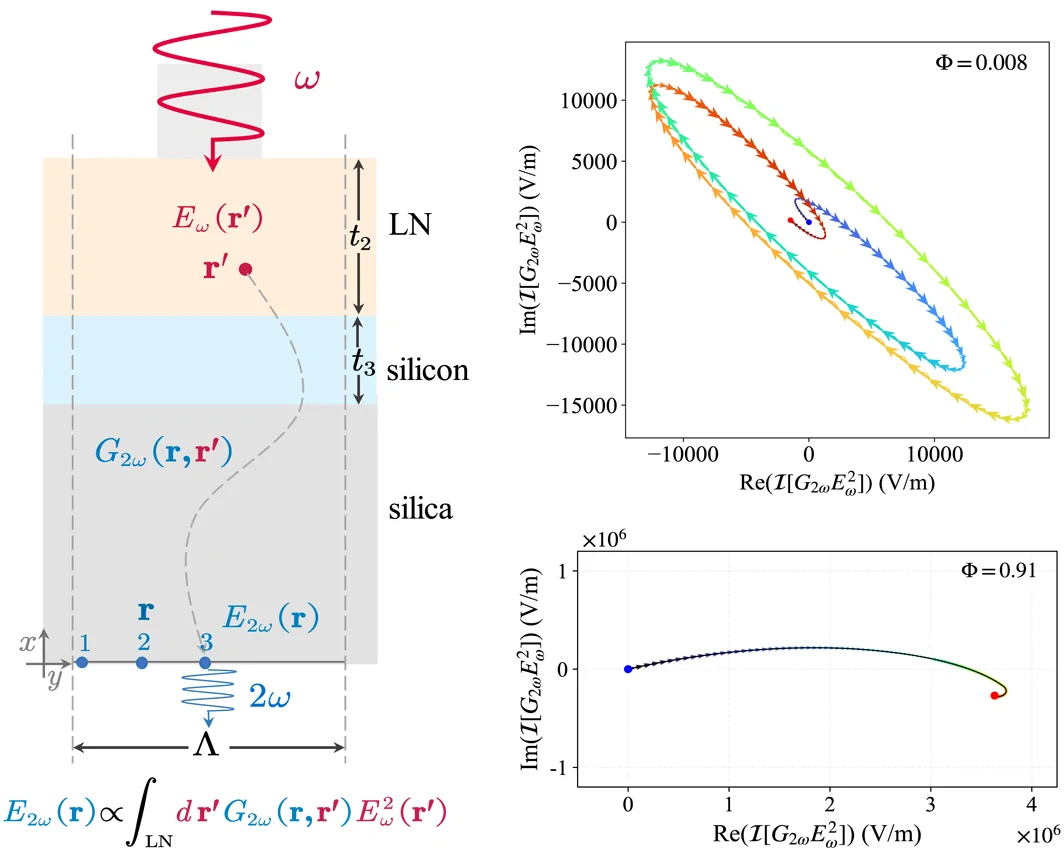

## 1 Introduction

Nonlinear optical frequency conversion originates from the nonlinear polarization of materials and underpins a wide range of applications in both classical and quantum optics, including integrated photonic circuits, ultrafast light sources, and quantum emitters [1, 2, 3, 4, 5]. Their efficiency (e.g., second‐harmonic generation, SHG) critically depends on satisfying the phase‐matching conditions, which ensure coherent buildup of the generated harmonic fields [6, 7, 8]. In bulk nonlinear media, phase matching is fundamentally limited by inherent wavevector mismatch caused by material dispersion [9, 10]. To overcome this constraint, several strategies have been developed, including birefringent phase matching, angle tuning to compensate for dispersion, and quasi‐phase matching based on periodic sign modulation of the nonlinear susceptibility [11, 12, 13, 14, 15]. These approaches enable constructive interference of the generated harmonics and maintain continuous energy transfer from the fundamental to the harmonic waves; however, conventional bulk materials are not well suited to the demands of integrated nonlinear photonics [16, 17, 18].

Micro/nanophotonic architectures have revolutionized nonlinear photonics by enhancing light–matter interactions through various resonant mechanisms to amplify fundamental field intensities [19, 20, 21, 22, 23]. In particular, the recent use of photonic structures supporting bound states in the continuum (BICs) with extremely high quality (*Q*) factors greatly enhances the SHG efficiency [24, 25, 26, 27, 28]. Actually, the complex phase distribution in nanoscale systems often gives rise to structure‐dispersion–induced phase mismatch that cannot be neglected [29, 30, 31]. A series of practical paradigms have been proposed, including the evaluation through mode‐overlap integration method in waveguides [26, 31, 32, 33, 34, 35, 36], as well as quasi‐phase‐matching strategies extended to twisted‐engineered two‐dimensional materials [14, 37]. Regardless, the conventional macroscopic phase‐matching framework fails to address the ubiquitous local phase mismatch present in subwavelength structures. Therefore, visualizing and optimizing microscopic phase‐matching processes in nonlinear nanophotonics provide an important perspective for enhancing nonlinear efficiency. Recently, we utilized the Green's function integration method (GFIM) for spatially resolved phase coherence analysis to quantitatively probe microscopic phase mismatch, achieving perfect phase matching [23]. Nevertheless, the bulky configuration of one‐dimensional distributed Bragg reflectors (DBRs) urges a shift toward two‐ and even three‐dimensional architectures to meet the demands of integrated nonlinear photonics.

Here, we extend the GFIM framework to two‐dimensional guide mode resonance (GMR) structures based on thin‐film lithium niobate (LN), and generalize the concept of phase‐matching factor (PMF). The PMF provides a quantitative measure of the spatial coherence of the constituent second‐harmonic (c‐SH) waves generated by microscopic nonlinear sources. The continuous destructive interference of c‐SH radiation in traditional GMR structures results in lower PMF, preventing any substantial improvement in SHG efficiency. Guided by the GFIM‐visualized c‐SH phasor ellipses, we propose several designs to improve the PMF. In particular, we introduce a high‐index waveguide layer to reshape and confine the fundamental field ($E_{\omega }$) to regions where the harmonic Green's function ($G_{2\omega }$) varies slowly. The resulting microscopic source, with intensity proportional to $G_{2\omega }E_{\omega }^{2}$, collectively generates a harmonic field whose constituent waves are nearly in‐phase. Consequently, the optimized GMR structure achieves a PMF exceeding 0.91 and a peak SHG conversion efficiency of 26.7% under a low pumping intensity of 2 kW/cm^2^. These findings demonstrate the capability of GFIM to capture complicated nonlinear interactions and provide a predictive phase‐engineered design framework for the rational design of compact, high‐performance integrated nonlinear optical devices.

## 2 Results and Discussion

### 2.1 Models and Methods

Lithium niobate‐on‐insulator (LNOI) combines strong $\chi^{\left(2\right)}$ nonlinearity, large electro‐optic coefficients, and low loss with tight optical confinement, forming an ideal platform for guided‐mode resonance enabling efficient SHG [3, 38, 39]. In our design (Figure 1a), an x‐cut LN film is bonded to a silicon dioxide substrate, on top of which a periodic polymethyl methacrylate (PMMA) grating is patterned. The grating grooves are aligned parallel to the c‐axis of LN (i.e., the z‐axis). Detailed geometric parameters and material properties are provided in Section 1 of Supporting Information. The periodic PMMA grating enables fully electron‐beam‐lithography‐fabricated devices, forming an all‐dielectric metasurface [40]. This perturbation layer diffracts the incident light, coupling it into the waveguide modes and forming leaky modes that lie above the light cone in momentum space (Figure 1b) [41, 42].

**FIGURE 1:**
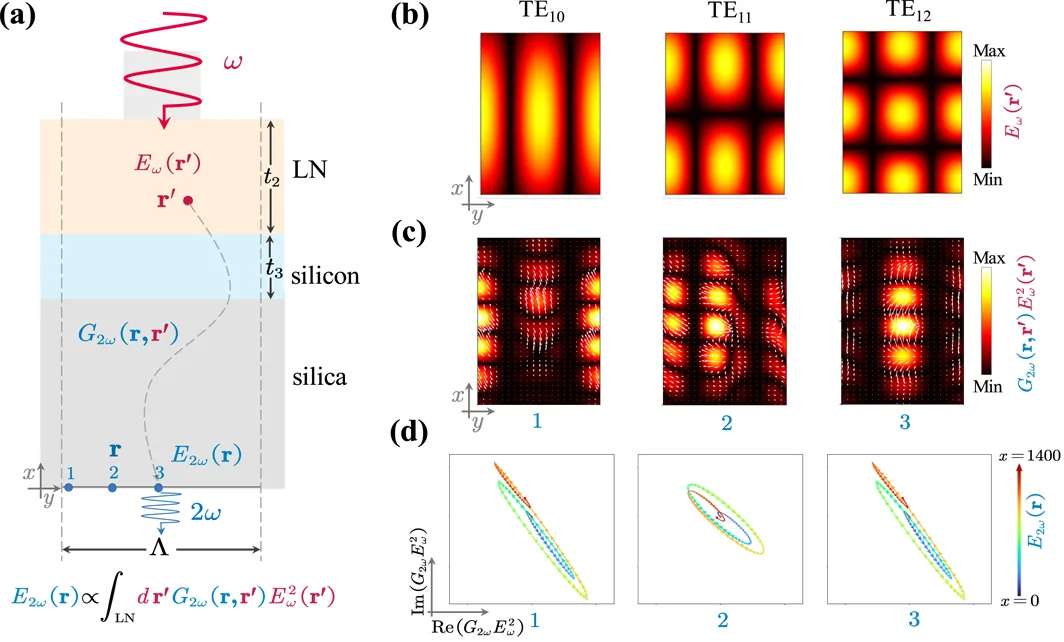
Visualizing SHG processes in a guided‐mode resonator using the Green's‐function integral method (GFIM). (a) Schematic of the SHG process in a lithium niobate‐based GMR structure. The structure consists of a periodic PMMA grating, a dual‐layer waveguide composed of both a linear (non‐$\chi^{\left(2\right)}$) and nonlinear (lithium niobate, $\chi^{\left(2\right)}$) layers, and a silica substrate. $t_{2}$ and $t_{3}$ denote the thicknesses of the two waveguide layers, whereas Λ corresponds to the structural pitch (Λ=1μm). (b–d) Using TE_10_ mode in conventional GMR ($t_{2}$ = 1400 nm and $t_{3}$ = 0) as an example, the SHG process is visualized by GFIM. (b) The incident fundamental wave excites GMR modes ($E_{\omega }$) TE_10_, TE_11_, and TE_12_ within the LN. (c) Spatial contribution of $G_{2\omega }E_{\omega }^{2}$ in LN for three representative radiation points (depicted in Figure 1a) under TE_10_ resonant excitation. Color denotes amplitude, and white arrows indicate the complex‐plane vectors. (d) The dipole amplitude scales as $G_{2\omega }E_{\omega }^{2}$, and the total second harmonic field $E_{2\omega }$ arises from the coherent superposition of the c‐SH waves from all microscopic nonlinear sources.

In nonlinear processes, the GFIM models the total second‐harmonic field by superposing the c‐SH contributions from distributed nonlinear polarization sources, thereby naturally capturing the SHG process [43]:

(1) $$E\left(2\omega ,r\right)=4\omega^{2}\mu_{0}\int dr^{\prime }G\left(2\omega ;r,r^{\prime }\right)\cdot P^{\left(2\right)}\left(2\omega ;r^{\prime }\right),$$

where $P^{\left(2\right)}\left(2\omega ;r^{\prime }\right)$ serves as the source term for SHG, and $G\left(2\omega ;r,r^{\prime }\right)$ is the harmonic Green's function, which quantifies the outcoupling efficiency between the harmonic source at the position $r^{\prime }$ within LN and the observation site at r (e.g., Figure 1c) [44]. To fully exploit the maximum nonlinear susceptibility of LN ($\chi_{33}^{\left(2\right)}$ = 30 pm/V), we employ a transverse‐electric (TE) polarized fundamental pump, such that only the out‐of‐plane polarization contributes [7]

(2) $$P_{2\omega }^{\left(2\right)}\left(x,y\right)=\varepsilon_{0}\chi_{33}^{\left(2\right)}E_{\omega }^{2}\left(x,y\right).$$

Moreover, the c‐SH field from an infinitesimal region within a nonlinear medium is proportional to the nonlinear polarization weighted by the Green's function:

(3) $$dE_{2\omega }\left(x,y,x^{\prime },y^{\prime }\right)\propto G_{2\omega }\left(x,y,x^{\prime },y^{\prime }\right)E_{\omega }^{2}\left(x^{\prime },y^{\prime }\right)dx^{\prime }dy^{\prime }.$$

Thus, the total SH field observed at the monitoring port, representing the coherent superposition of all c‐SH contributions (Figure 1d), can be expressed as follows:

(4) $$E_{2\omega }\left(x,y\right)=\frac{4\omega^{2}\chi_{33}^{\left(2\right)}}{c^{2}}\iint_{\mathrm{LN}}dx^{\prime }dy^{\prime }G_{2\omega }\left(x,y,x^{\prime },y^{\prime }\right)E_{\omega }^{2}\left(x^{\prime },y^{\prime }\right).$$

The complex‐valued integrand $G_{2\omega }E_{\omega }^{2}$ phasor determines the interference behavior of the microscopic contributions. Efficient SHG requires the phase of $G_{2\omega }E_{\omega }^{2}$ to remain consistent over the nonlinear region, ensuring constructive interference; otherwise, phase variations lead to destructive interference and reduced conversion efficiency. To quantify the spatial coherence of c‐SH waves at a given monitoring port x, we define the pointwise phase‐matching factor (pPMF) at any point y as follows:

(5) $$\Phi \left(x,y\right)=\frac{\left|\iint_{\mathrm{LN}}G_{2\omega }\left(x,y;x^{\prime },y^{\prime }\right)E_{\omega }^{2}\left(x^{\prime },y^{\prime }\right)dx^{\prime }dy^{\prime }\right|}{\iint_{\mathrm{LN}}\left|G_{2\omega }\left(x,y;x^{\prime },y^{\prime }\right)E_{\omega }^{2}\left(x^{\prime },y^{\prime }\right)\right|dx^{\prime }dy^{\prime }}.$$

This dimensionless factor quantifies the degree of constructive interference among all c‐SH contributions and is independent of the absolute pump amplitude. A value Φ=1 corresponds to perfect phase matching, an ideal condition rarely achieved due to intrinsic spatial phase variations in the complex product $G_{2\omega }E_{\omega }^{2}$ throughout the nonlinear medium.

More complex than one‐dimensional case, the SH intensity at the monitoring port can vary significantly across the port. To evaluate the degree of phase coherence over the entire port located at x, we introduce the overall phase‐matching factor (oPMF or simply PMF) as follows:

(6) $$\Phi (x)=\frac{\left|\int_{-\Lambda ⁄2}^{\Lambda ⁄2}dy\iint_{\mathrm{LN}}G_{2\omega }\left(x,y;x^{\prime },y^{\prime }\right)E_{\omega }^{2}\left(x^{\prime },y^{\prime }\right)dx^{\prime }dy^{\prime }\right|}{\int_{-\Lambda ⁄2}^{\Lambda ⁄2}dy\iint_{\mathrm{LN}}|G_{2\omega }\left(x,y;x^{\prime },y^{\prime }\right)E_{\omega }^{2}\left(x^{\prime },y^{\prime }\right)|dx^{\prime }dy^{\prime }},$$

where Λ denotes the grating period. This metric captures global constructive interference of all c‐SH contributions across the port. By contrast, the arithmetic mean of the local phase‐matching factor, $\Phi \left(x\right)=\frac{1}{\Lambda }\int_{-\Lambda /2}^{\Lambda /2}\Phi \left(x,y\right)dy,$ only reflects the average spatial coherence and neglects interference effects encoded in the numerator of Equation (6). The pPMF provides a spatially resolved measure of phase coherence, whereas oPMF offers a more comprehensive evaluation for analyzing nonlinear interactions in complex nanostructured systems.

### 2.2 Phase Mismatch in Conventional GMR Structure

We first examine how resonance modes evolve with nonlinear‐layer thickness (1100–1400 nm) in a conventional GMR structure, motivated by the potential of resonant pumping to enhance nonlinear interactions. The transcendental dispersion relation (Equation S11) for a four‐layer planar waveguide is used to determine the photonic bands of both conventional and optimized GMR structures (Figure S1). Eigenmode analysis using COMSOL confirms these as three TE waveguide modes, TE_10_, TE_11_, and TE_12_ (Figure S2a). The linear spectrum provides the foundation for understanding enhanced light–matter interactions in GMR systems (Figure S2b). Among them, the TE_10_ mode exhibits the strongest field confinement and the highest quality factor, thus is the most favorable candidate for efficient nonlinear conversion (Figure S2c).

In nonlinear aspect, the SHG efficiencies of TE_10_ (Figure S3a) and TE_12_ (Figure S3c) modes increase with the LN thickness. To quantify the correlation between fundamental fields $E_{\omega }$ and SHG efficiency, we define a field enhancement factor $\bar{E_{\text{norm}}^{2}}$

(7) $$\bar{E_{\text{norm}}^{2}}=\frac{\int_{\mathrm{LN}}|E_{\omega }\left(x,y\right){|}^{2}dS}{\int_{\mathrm{LN}}|E_{\text{in}}\left(x,y\right){|}^{2}dS},$$

where $E_{\text{in}}$ is the incident field amplitude. Using $\bar{E_{\text{norm}}^{2}}$ as a metric, we observe a strong, though not strictly linear, correlation between field enhancement and SHG efficiency for the TE_10_ and TE_12_ modes. In contrast, the SHG efficiency of TE_11_ mode decreases with increasing $\bar{E_{\text{norm}}^{2}}$ (Figure S3b and Figure 2a). These anomalies suggest that SHG efficiency involves factors beyond field enhancement (Figure 2b).

**FIGURE 2:**
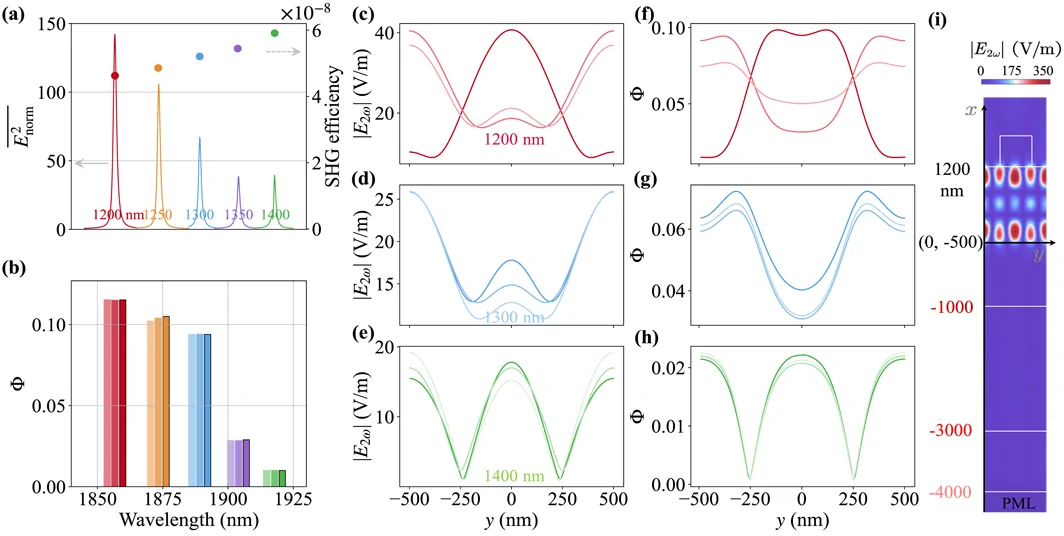
Phase‐matching‐factor analysis of the anomalous SHG in TE_11_ mode. (a) Field enhancement factor $\bar{E_{\text{norm}}^{2}}$ (left axis, solid dots) and SHG efficiency (right axis, solid line) for the TE_11_ mode pumping versus LN thickness ($t_{2}$ = 1200–1400 nm). (b) Overall phase‐matching factor (PMF, Φ) at resonance as a function of LN thickness. (c–e) Harmonic intensity profiles along the y‐axis at different three radiation ports for $t_{2}$ = 1200 nm (b), 1300 nm (c), and 1400 nm (d). (f–h) Corresponding pointwise PMF for the same thicknesses. (i) Harmonic field distribution under TE_11_ mode‐pumping with an incident power density of 2 kW/cm^2^ ($t_{2}$ = 1200 nm). Perfectly matched layers (PMLs) are applied above the excitation source and contact with far‐field port in simulations. Curves (columns) with varying transparency denote three different detection ports, with the farthest port corresponding to the most transparent color. Calculations across different detection ports show that pPMF (Equation (5)) accurately captures any local phase matching, whereas oPMF (Equation (6)) provides a robust measure of the overall phase‐matching quality.

As the radiated harmonic field deviates from a plane wave (Figure 2c–e, Figure S4), we examine the pointwise PMF of the TE_11_ mode along y at three representative output ports (Figure 2f–h). Taking the case of $t_{2}$ = 1200 nm as an example, the harmonic field distribution is shown in Figure 2i. The pointwise values $\Phi \left(x_{1},y\right)$ at $x_{1}$ = −1000 nm remain low, and exhibit strong variation across y, differing by up to a factor of 6. As the observation port approaches the far‐field (contact with the perfect matching layer, PML), the x‐dependence of Φ(x,y) becomes less significant, while noticeable transverse variation persists. For other LN thicknesses, similar x and y‐dependence is observed, with Φ(x,y) consistently low. Despite minor deviations, the pointwise PMF Φ(x,y) captures local phase matching at any radiation point.

The overall PMF yields consistent values across all three ports for different LN thicknesses (Figure 2b), demonstrating that it effectively captures the global phase‐matching behavior. This consistency likely stems from the conservation of second‐harmonic power across output channels. The oPMF thus provides a convenient and reliable metric for evaluating overall phase‐matching quality, and all subsequent analyses are based on it. This PMF peaks at $t_{2}$ = 1200 nm despite the weakest field enhancement, whereas at $t_{2}$ = 1400 nm, stronger field enhancement coincides with a pronounced PMF reduction. The inverse PMF–field correlation shows that poor phase matching limits energy conversion from fundamental to harmonic waves. Moreover, the PMF values below 0.16 in all three modes strongly restrict SHG efficiency (Figure S3d–f), highlighting the need to capture microscopic local phase mismatch.

We perform the detailed analysis on TE_10_ resonant mode, which exhibits the strongest field amplitude but the lowest PMF Φ (Figure S3). As shown in Figure 3a, the normalized field intensity exceeded 40 ($\bar{E_{\text{norm}}^{2}}$ = 598) at the resonance wavelength 2119.32 nm, in a 1400‐nm‐thick LN waveguide. Within the nonlinear region, TE_10_ assumes two distinct phase states per period, differing by π, with two field nodes (Figure 3b). Consequently, the phase of $E_{\omega }^{2}$ remains constant, and the overall phase of the product $G_{2\omega }E_{\omega }^{2}$ is determined solely by the Green's function $G_{2\omega }$. This behavior is consistent with the resonance‐induced phase‐locking observed previously in cavity‐integrated one‐dimensional structures [23].

**FIGURE 3:**
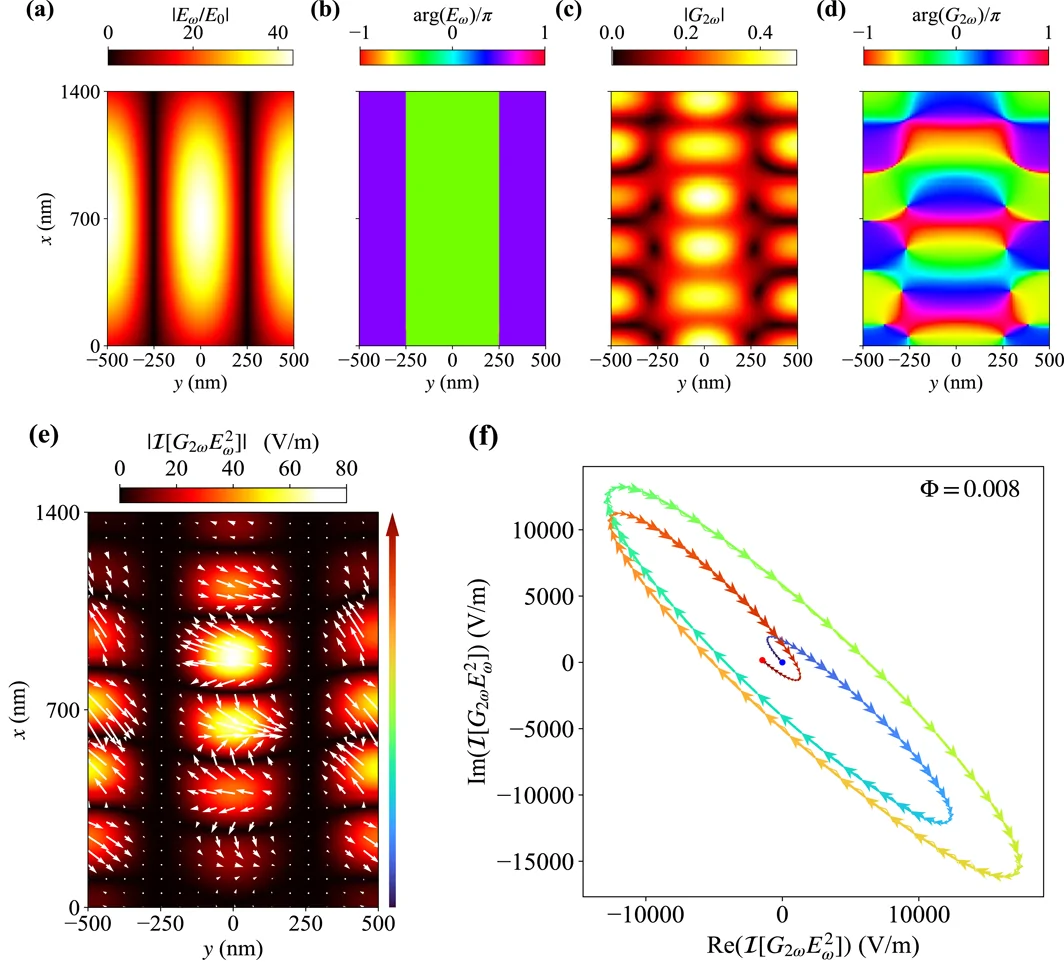
Microscopic phase‐mismatch analysis using the GFIM. (a, b) Amplitude (a) and phase (b) distributions of the fundamental TE_10_ mode at $t_{2}$ = 1400 nm. A phase discontinuity by π occurs across the field node along the y‐axis, leading to a uniform phase distribution of $E_{\omega }^{2}$. (c, d) Amplitude (c) and phase (d) of the Green's function at the center of the detecting port at x = −4000 nm. (e) Distribution of the product $G_{2\omega }E_{\omega }^{2}$ after integration along the y‐direction at the port. White arrows denote the vector representation of $I\left[G_{2\omega }E_{\omega }^{2}\right]$. (f) Sequential head‐to‐tail connection of these vectors along the x‐axis (from blue to red) illustrates the superposition process of $I\left[G_{2\omega }E_{\omega }^{2}\right]$. Each arrow represents the cumulative effect over a layer. Continuous destructive interference of the $I\left[G_{2\omega }E_{\omega }^{2}\right]$ yields the elliptical trajectory, resulting in an overall PMF of only 0.008.

As shown in Figure 3c–d, the Green's function to the port center (y = 0) oscillates nearly periodically in both amplitude and phase, forming multiple cycles within the LN waveguide. The pronounced variation of $G_{2\omega }E_{\omega }^{2}$ maps across different locations (Figure S5) indicates that microscopic phase mismatch at any single point can be reliably inferred. Meanwhile, to capture the overall contribution across the entire port, we evaluate the line integral

(8) $$I\left[G_{2\omega }E_{\omega }^{2}\right]=\int_{\text{Portline}}G_{2\omega }\left(x,y\right)E_{\omega }^{2}\left(x,y\right)dy,$$

and visualize its magnitude and complex phasor in Figure 3e. This integrated phasor coherently accounts for the collective radiation toward all port positions. Due to the steep spatial phase variation of $G_{2\omega }$, adjacent maxima in $\left|I\left[G_{2\omega }E_{\omega }^{2}\right]\right|$ may possess nearly opposite phases (≈π shift) in $I\left[G_{2\omega }E_{\omega }^{2}\right]$, leading to destructive interference among c‐SH waves (Figure S6).

We further visualize the vector summation of $I\left[G_{2\omega }E_{\omega }^{2}\right]$ across the entire LN region, with each phasor's argument given by the $I\left[G_{2\omega }E_{\omega }^{2}\right]$ phase. Column‐wise integration produces highly disordered phasor accumulation patterns (Figure S7), while row‐wise summation generates approximately elliptical trajectory in the complex plane (Figure 3f), although the two methods are equivalent. Certain segments along the major axis exhibit large local magnitudes; yet, they are largely canceled by phase‐opposed contributions elsewhere along the ellipse. Consequently, the net harmonic amplitude remains small, with an overall PMF of only 0.008. These results suggest that harnessing locally constructive regions could provide an effective route to boost nonlinear conversion in otherwise phase‐mismatched photonic systems.

### 2.3 Optimization Design Guided by Phase Analysis

Beyond mode engineering, approaches, such as wavefront shaping and structured‐light illumination, have been demonstrated to tailor the phase of the fundamental field and thereby enhance phase matching [26, 45, 46]. Leveraging the simplicity of angle tuning, we employ oblique incidence to reshape the fundamental field. As shown in Figure S8, despite a noticeable decrease in field enhancement, the PMF markedly increases from 0.008 to 0.13 at an incidence angle of 41.95°, resulting in nearly unchanged SHG efficiency. Nevertheless, the near‐periodic radiative nature of the Green's function in conventional GMR structures ultimately constrains performance. Under nonresonant pumping, mode tuning can, in principle, yield better phase matching (e.g., a PMF of 0.82 for a fundamental wavelength of 2000 nm), but the reduced field strength suppresses SHG efficiency; we therefore do not pursue this scenario further.

To extend the analysis, we apply the GFIM framework to reported nonlinear photonic structures, exemplified by quasi‐BIC modes in the period‐doubled GMR configurations [47]. The phasor summation of $I\left[G_{2\omega }E_{\omega }^{2}\right]$ forms an approximate half‐ring pattern (Figure S9), analogous to half of the elliptical trajectory shown in Figure 3f, leading to a substantial increase in PMF up to 0.28. This improvement primarily arises from restricting the Green's function variation to within one period via reduced LN thickness, combined with resonance‐induced field enhancement.

Motivated by the partial phasor summation along the elliptical trajectory that enhances the PMF (Figure 3f and Figure S9), we optimize phase matching by replacing the substrate‐adjacent region with a refractive‐index‐matched non‐$\chi^{\left(2\right)}$‐active material. In Figure 4a, by preserving the TE_10_ profile (keeping $t_{2}+t_{3}$ = 1400 nm), this modification suppresses destructive interference by removing opposite‐phase contributions near the substrate. We track the TE_10_ mode field enhancement factor $\bar{E_{\text{norm}}^{2}}$, SHG efficiency, and overall PMF as functions of non‐$\chi^{\left(2\right)}$ thickness $t_{3}$ (Figure 4b–d). The field enhancement exhibits a nonmonotonic trend, rising slightly before decreasing, whereas the PMF oscillates and approaches unity as $t_{3}$ approaches the total thickness. The SHG efficiency reflects a balance among the nonlinear interaction volume, the local field enhancement, and the phase‐matching conditions. At $t_{3}$ = 590 nm, the SHG efficiency reaches its maximum of 6.8 × 10^−6^ (40‐fold enhancement), corresponding to an overall PMF of 0.21 (26‐fold increase) and a nearly unchanged $\bar{E_{\text{norm}}^{2}}$ (1.05‐fold). This indicates that phase matching is the key factor determining the SHG efficiency in the absence of field enhancement in nanostructures. In ultrathin nonlinear layers, SHG efficiency drops below 10^−9^ due to the vanishing interaction volume, even though the PMF approaches its theoretical maximum. This explains why phase mismatch is often neglected in atomically thin materials.

**FIGURE 4:**
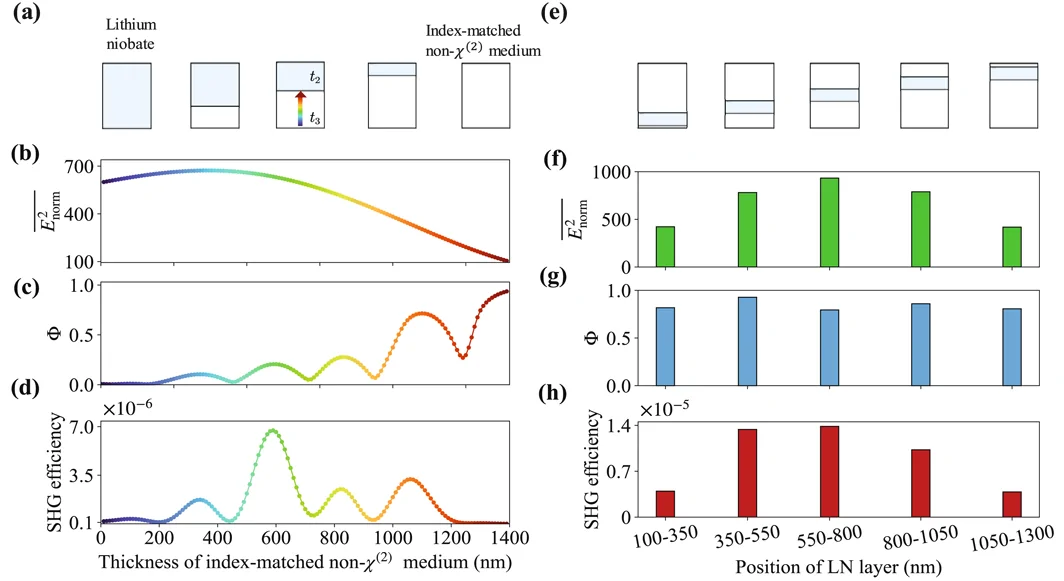
Two strategies for optimizing the phase‐matching factor based on superposition trajectory. (a) Schematic of a structure where the nonlinear layer is partially replaced by refractive‐index‐matched non‐$\chi^{\left(2\right)}$ dielectric material. (b–d) Field enhancement factor (b), PMF (c), and SHG efficiency (d) as a function of non‐$\chi^{\left(2\right)}$ material thickness. (e) Schematic of localized LN incorporation to support constructive interference. (f–h) Field enhancement factor (f), PMF (g), and SHG efficiency (h) for various designs. PMF remains consistently high (> 0.75) over a broad parameter range, whereas SHG efficiency follows the trend of field enhancement owing to favorable phase matching.

We further selectively retain the inner 200–250 nm of the LN layer (blue), corresponding to the elliptical phasor arc that contributes predominantly constructive interference in the complex plane (Figure 4e). The remaining region (white) is replaced with a refractive‐index‐matched non‐$\chi^{\left(2\right)}$‐active material to preserve the TE_10_ mode. A fabrication‐compatible silicon nitride can replace the refractive‐index‐matched non‐$\chi^{\left(2\right)}$ material layer [26]. In this configuration, both a relatively high PMF (Φ > 0.75) and a significantly enhanced SHG efficiency of 10^−5^ are achieved (Figure 4f–h). Remarkably, reducing the nonlinear layer to only 200–250 nm doubles the SHG efficiency compared with the previous 600 nm optimized design (Figure 4d). These results show that the phasor‐trajectory metric reliably identifies spatial regions that contribute constructively to SHG, enabling targeted placement of nonlinear polarization sources for improved performance.

### 2.4 Silicon–Lithium Niobate Dual Waveguide Design for Simultaneous Field and Phase Matching Optimization

To further enhance the field effect, we replace the previously index‐matched non‐$\chi^{\left(2\right)}$ layer with the high‐refractive‐index silicon waveguide ($t_{3}\neq 0$ in Figure 1). After parametric optimization, we selected a 400 nm silicon layer thickness (Figure S10). Figure 5a compares the field enhancement factor $\bar{E_{\text{norm}}^{2}}$, phase‐matching factor Φ, and SHG efficiency for the dual‐waveguide configurations. The normalized field amplitude in LN increases to 380 (Figure 5b), corresponding $\bar{E_{\text{norm}}^{2}}$ reaches 10^4^—a 20.6‐fold improvement over the silicon‐free design. This enhancement arises from strong TE_10_ confinement in silicon layer, with its evanescent tail efficiently coupling into the nonlinear LN. Importantly, the phase distribution of the fundamental field remains unchanged under resonant condition (Figure 5c), so the phase of c‐SH waves continues to be governed by the Green's function (Figure 5d,e). Since the enhanced field is now concentrated in a thinner LN section where $G_{2\omega }$ exhibits a slowly varying spatial phase, the phasor values of $I\left[G_{2\omega }E_{\omega }^{2}\right]$ (indicated by white arrows) become nearly aligned in regions of large magnitude (Figure 5f and Figure S11). Row‐wise accumulation of phasors across the LN layer affords a smooth trajectory with a slightly curled tail in the complex plane (Figure 5g), indicating enhanced constructive interference compared with the case in Figure 3f. Finally, the PMF reaches 0.63, and the SHG efficiency rises to 6.5 × 10^−3^, corresponding to a 3.82 × 10^4^‐fold improvement over the original structure, and a 465‐fold improvement relative to the design with a partially preserved LN layer in Figure 4h (the middle sample).

**FIGURE 5:**
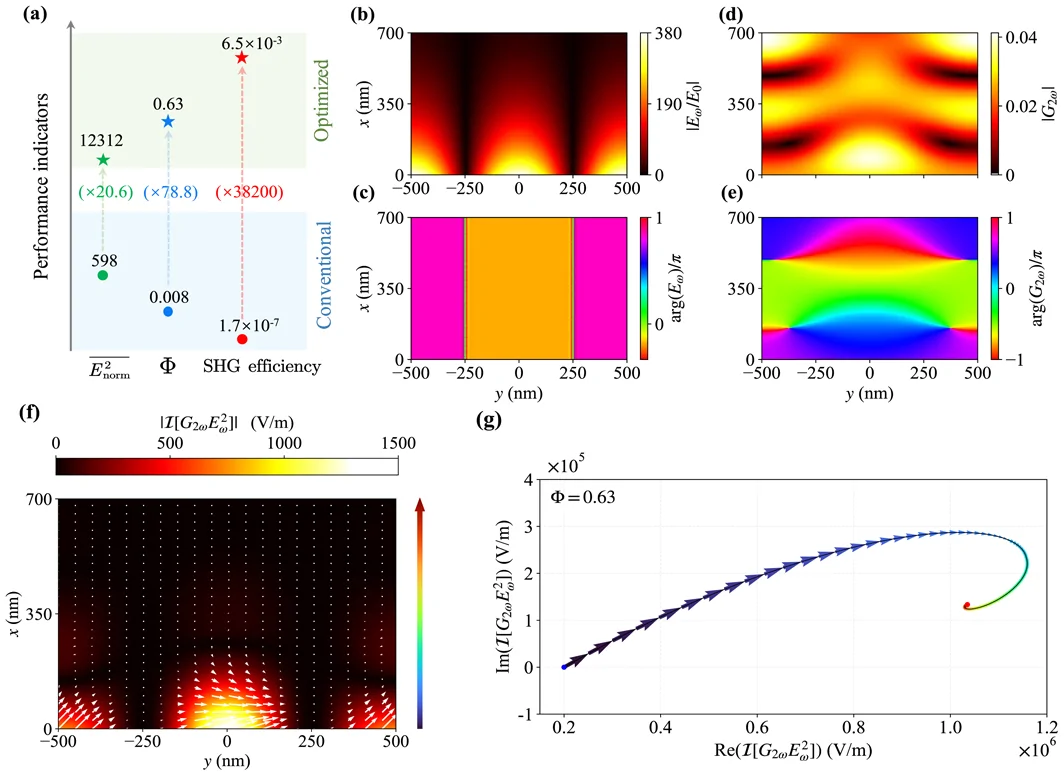
Phase‐matching analysis of the optimized dual‐waveguide GMR structure. (a) Comparison of key performance metrics between the optimized ($t_{2}$ = 400 nm and $t_{3}$ = 700 nm) and the conventional ($t_{2}$ = 1400 nm and $t_{3}$ = 0) GMR structure. Values in parentheses denote the enhancement factors relative to the conventional configuration. (b–c) Amplitude (b) and phase (c) distributions of the fundamental TE_10_ mode in the LN layer. (d–e) Amplitude (d) and phase (e) of the Green's function to the center of the output port. (f) Amplitude distribution of $I\left[G_{2\omega }E_{\omega }^{2}\right]$, primarily concentrated in regions where the phase of Green's function varies slowly. (g) Vector superposition of the phasor $I\left[G_{2\omega }E_{\omega }^{2}\right]$, revealing a favorable phase matching picture.

To assess the robustness of phase matching in the optimized GMR configuration, we examine how variations in LN and silicon layer thicknesses affect SHG efficiency (Figure S10). Under favorable phase‐matching conditions (all PMF > 0.6), increasing either layer thickness enhances SHG efficiency through stronger field confinement. In particular, a modest increase in LN thickness boosts the SHG efficiency up to 2.75%. Beyond layer thickness, the fundamental field in the LN can be further amplified by modulating the duty cycle of the PMMA gratings. Rigorous coupled‐wave analysis (RCWA) [48] shows that the resonance peak remains near 2883.5 nm as the PMMA duty cycle increased from 0.1 to 0.9, whereas the full‐width at half‐maximum narrows at both extremes, indicating a higher *Q* factor (Figure 6a). Correspondingly, at a duty cycle of 0.9, the maximum field enhancement factor and SHG efficiency increase by factors of 6 and 40, respectively (Figure 6b). At a low pump intensity of 2 kW/cm^2^, the optimized structure reaches 26.7% SHG efficiency, exceeding previous reports by over an order of magnitude (Table 1). This value is computed from a coupled model that accounts for pump depletion (Figure S12). Under robust phase matching, optimizing field enhancement enables highly efficient frequency conversion even at low pump intensities.

**FIGURE 6:**
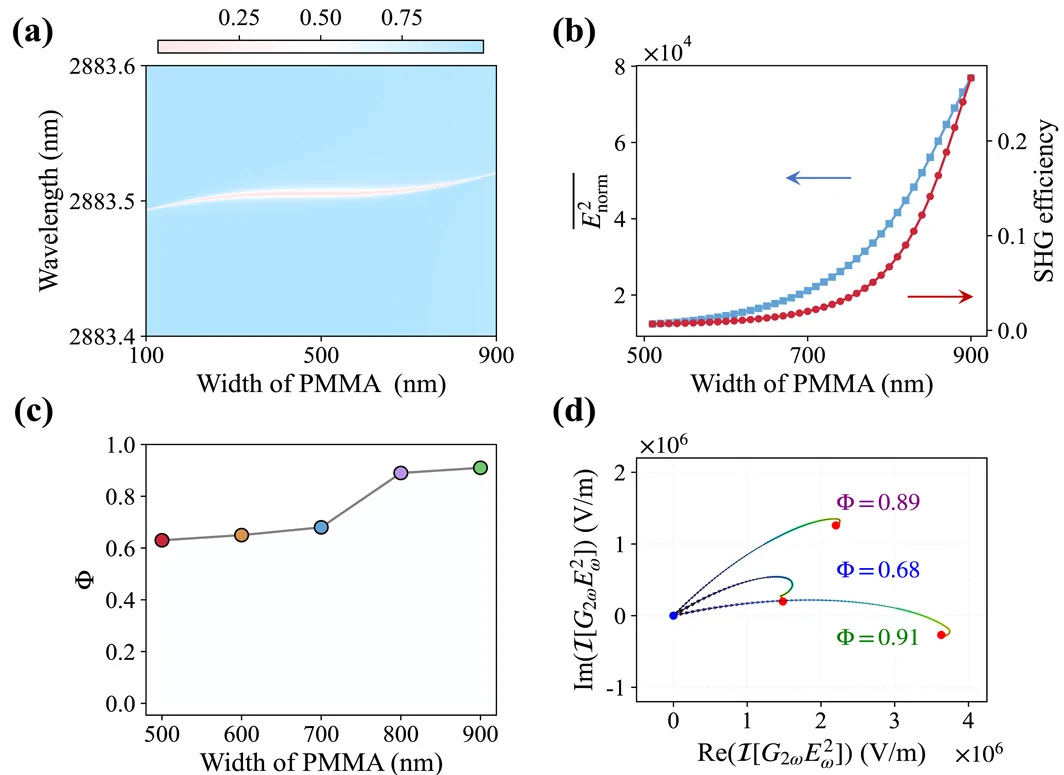
Optimization of SHG efficiency via optimized field enhancement and phase‐matching. GFIM analysis with varying PMMA width shows that the optimized phase‐matching factor enhances the effectiveness of the field enhancement, leading to a breakthrough in SHG efficiency. (a) Transmission spectra versus fundamental wavelength and PMMA width. (b) Field enhancement factor $\bar{E_{\text{norm}}^{2}}$ (left axis, blue) and SHG efficiency (right axis, red) as functions of PMMA width. (c) Phase‐matching factor varies with the duty cycle of the PMMA gratings. (d) Vector superposition of $I\left[G_{2\omega }E_{\omega }^{2}\right]$ for PMMA widths of 700 (blue), 800 (purple), and 900 nm (green), showing that higher field enhancement increases the fraction of constructive interference of the c‐SH waves generated by microscopic nonlinear sources, thereby improving the phase‐matching factor.

**TABLE 1:** Comparison of SHG efficiency enhancement in different LN‐based structures.

Structure	Input power (MW/cm^2^)	SHG efficiency	Reported year	References
LN waveguide GMR	1.3 × 10^3^	8.1 × 10^−5^	2021	[17]
LN on Bragg reflector	1.3 × 10^3^	6.0 × 10^−3^	2024	[20]
Two‐layer LN metasurface	5.3 × 10^3^	1.4 × 10^−4^	2021	[39]
LN waveguide GMR	4.0 × 10^−1^	5.0 × 10^−2^	2022	[28]
LN metasurface	3.3 × 10^−3^	4.9 × 10^−3^	2021	[24]
LN photonic crystal slab	2.0 × 10^−3^	1.0 × 10^−2^	2023	[18]
LN photonic crystal slab	2.0 × 10^−3^	3.7 × 10^−2^	2023	[16]
LN waveguide GMR	5.0 × 10^4^	1.36 × 10^−3^	2025	[40]
LN photonic crystal slab	2.0 × 10^−3^	2.26 × 10^−2^	2025	[38]
LN waveguide GMR	1.0 × 10^0^	2.8 × 10^−2^	2023	[27]
LN and silicon dual‐waveguide GMR	2.0 × 10^−3^	2.67 × 10^−1^		**Our work**

We display the process of microscopic phase matching. At a duty cycle of 0.9, the harmonic field evolves toward a plane‐wave profile away from the grating, causing the pointwise PMF at the output port to converge to the overall PMF (Figure S13). The PMF increases from 0.63 to 0.91 (Figure 6c), approaching near‐perfect phase matching. It confirms that the observed SHG enhancement cannot be attributed solely to the fundamental‐field amplification. Figure 6d further illustrates that the improved phase matching shortens the curled tail of phasor trajectory of $I\left[G_{2\omega }E_{\omega }^{2}\right]$, yielding favorable constructive interference. Therefore, field enhancement sets the theoretical upper bound of SHG efficiency, whereas phase matching dictates the practically achievable limit, serving as a decisive lever for engineering high‐performance nonlinear photonic structures.

## 3 Conclusions and Outlook

In summary, we extend the Green's function integral method to visualize and optimize phase matching in LN‐based GMR structures. We reveal that the counterintuitive decrease of SHG efficiency with increasing field enhancement in conventional GMRs arises from severe phase mismatch. Guided by the phasor‐summation picture provided by GFIM, we introduce a high‐index waveguide layer that redistributes the fundamental field into regions where the harmonic Green's function varies slowly, achieving a PMF exceeding 0.91 and a high SHG conversion efficiency of 26.7% under a low pump intensity of 2 kW/cm^2^.

These findings establish the phase‐matching factor as a decisive design metric that complements conventional field‐enhancement considerations, enabling predictive and phase‐engineered strategies for various nonlinear nanophotonic devices. Looking forward, the GFIM framework can be generalized to fully three‐dimensional architectures, higher‐order and difference‐frequency generation, as well as non‐Hermitian and topological photonic platforms where coherent superposition is essential. Moreover, since the GFIM inherently captures information on coherent interaction, it may also provide a valuable tool for quantum photonic platforms where coherence must be precisely controlled or intentionally suppressed.

## 4 Numerical Simulation Method

We validated the Green's function method via finite‐element simulations in COMSOL. Under the undepleted‐pump approximation, the fundamental and harmonic fields are solved sequentially, with the nonlinear polarization driving the SH field. For pump depletion, the mutual coupling between fundamental and harmonic fields is fully accounted for via self‐consistent solution of the coupled‐wave equations. The total SHG power is obtained by integrating the time‐averaged Poynting vector of the radiated SH field at output port. To compute the Green's function describing radiation from a unit‐area polarization source ($J_{z}=-j\omega P_{z}$), an out‐of‐plane current is swept across the LN region. This procedure directly maps the system's linear response, establishing a clear link between the fundamental field, harmonic Green's function, and generated SH output.

## Author Contributions

**Chengkang Liang:** software, methodology, conceptualization, investigation, validation, formal analysis, visualization, writing – original draft, writing – review and editing. **Quanying Li:** software, writing – review and editing. **Jiale Xu:** software, writing – review and editing. **Pingqi Gao:** funding acquisition, project administration, resources. **Jiancan Yu:** conceptualization, methodology, investigation, validation, software, formal analysis, data curation, supervision, funding acquisition, visualization, writing – review and editing, writing – original draft, project administration.

## Supporting information


Supporting Information S1

## Data Availability

The data that support the findings of this study are available from the corresponding author upon reasonable request.
