# Supplementary material for: Visualizing and Optimizing Phase Matching in Nonlinear Guided‐Mode Resonators With the Green's Function Integral Method
Source: Nanophotonics. 2026 Apr 20;15(8):e70091. doi: 10.1002/nap2.70091 (PMC13642535; doi:10.1002/nap2.70091)
Supplement: Supplementary file 1 [file NAP2-15-e70091-s001.docx]

**Supporting Information:** **Visualizing and Optimizing Phase Matching in Nonlinear Guided-Mode Resonators with the Green’s Function Integral Method**

*Chengkang Liang**,^1^ Quanying Li,^1^ Jiale Xu,^1^ Pingqi Gao**,^1^ and Jiancan Yu^1,*^*

^1^School of Materials, Sun Yat-sen University, Shenzhen, China, 518107

*yujc3@mail.sysu.edu.cn

1. **Dispersion relationship of double-layer waveguide model**

To exploit the guided-mode resonance (GMR) for enhancing the fundamental field and thereby achieving higher second-harmonic generation (SHG) efficiency, we first analyze the dispersion relation of the transverse electric (TE) mode in a more comprehensive four-layer planar waveguide structure, as shown in Fig. S1. Treating periodic PMMA grating as perturbation (), the structure consists of air (), lithium niobate (LN) slab (), Si film () and semi-infinite thickness silica substrate (). Under the-polarized light incidence, the planar transverse electric wave appears in the form of(*j* = 0, 2, 3, 4), and represents the propagation constant ^[1]^

where is the incident angle, is the virtual pitch, and is the diffraction order. Electric field mode in each layer of material satisfies the wave equation

where, is the angular frequency and is the light speed in vacuum. Specifically, the distribution of electric field in each layer of the four-layer waveguide is described as:

where represent their amplitudes. Substituting Eq. into Eq. , we get the detailed wavevector components:

By applying the continuity condition of electric field boundary at, 0,, we obtain:

Therefore, we can rewrite the expression for the electric fields:

The magnetic field is found using Ampere’s law as, with the in-plane component given by:

Similarly, the continuity of the magnetic field applied at boundary, 0,:

Combining these equations, we obtain

which yields:

Rearranging, we obtained the general TE dispersion equation that explains the optimized GMR model

As the thickness of the silicon layer approaches zero, the optimized GMR described in Eq. degenerates into the dispersion relation of the three-layer conventional GMR model ^[2]^:

**Fig. S1.**  Effective model of proposed GMR structure, consisting of a dual-layer waveguide (non-silicon and lithium niobate), simplified into a four-layer planar structure.

2. **Supporting Figures**

**Fig. S2**. **Linear** **optical characteristics of a conventional GMR structure with a single-layer LN waveguide configuration** (). (a) Dispersion relations of the three resonant modes as a function of the LN thickness (1100-1400 nm), obtained by solving the transcendental equation Eq. . The inset shows the corresponding electric field eigenmode obtained from COMSOL eigenmode analysis. (b) Mode-resolved spectral responses calculated using rigorous coupled-wave analysis, showing the evolution of the three modes with varying LN thickness (represented by different color). (c) Quality -factors (Q-factors) of the three modes extracted via Fano fitting ^[3]^, indicating strong light–matter interaction in the GMR structure. We focus primarily on the fundamental low-frequency transverse electric TE_10_ mode, which exhibits the highest Q-factor.

**Fig. S3. Nonlinear analysis of conventional GMR structure.** (a-c) SHG efficiencies (right axis, solid line) and field enhancement factors (left axis, dots) of three modes, TE_10_ (a), TE_11_ (b) and TE_12_ (c), and (d-f) corresponding overall phase-matching factors, are plotted as functions of LN thickness (1100-1400 nm). For the TE_10_ mode, the SHG efficiency (a, right axis, solid line) is determined by both the field enhancement factor and the overall PMF (d). Notably, the abnormal behavior of SHG efficiency, which does not increase monotonically with field enhancement, indicates that the overall PMF plays a critical role—highlighting the importance of phase matching in nanophotonic structures. Panels (b, e) and (c, f) show results for the TE_11_ (middle column) and TE_12_ (rightmost column) modes, respectively, with (b) and (c) depicting their field enhancement and SHG efficiency, and (e) and (f) showing the corresponding overall PMFs. Lower-order modes exhibit stronger field confinement and higher SHG efficiency, whereas their phase-matching factors are generally smaller than those of higher-order modes.

**Fig. S4.** **Validation of the Green’s function method for second-harmonic field calculation.** (a-c) Comparison of the second-harmonic field amplitude of for TE_11_ mode obtained from the Green’s function method (red dot) and finite element simulations (black line) for different LN thicknesses: (a) = 1200 nm, (b)= 1300 nm, and (c) = 1400 nm.  The fundamental field distribution within the lithium niobate layer is recorded through full-wave simulations in COMSOL. The general approach to simulating second harmonic generation in COMSOL Multiphysics, under the undepleted-pump approximation, is to treat the nonlinear polarization at the harmonic wavelength as a source in a separate linear simulation to evaluate the system’s nonlinear response. While the second-harmonic field is computed by Green’s function method as follows. First, by sweeping out-of-plane current sources at the harmonic frequency across the entire LN region, we obtained the Green’s function that characterizes the radiation into the ports from a unit-area polarization located at any position within the nonlinear medium, where the simulated linear field amplitude of the current sources corresponds to the polarization amplitude per unit area. Then, the second-harmonic field is obtained by integrating the product of the Green’s function, which represents the out-of-plane radiation from a current source at to the observation point, and the recorded fundamental field over the entire lithium niobate region, as described by. The output here comes from the farthest port as shown in Fig. S5a. The primary source of error arises from spatial misalignment between the field of fundamental wave and the harmonic Green’s function.

**Fig. S5. Phase-matching analysis of a conventional GMR structure at the representative positions at the output port for the TE_10_ resonant mode.** The LN waveguide thickness = 1400 nm. (a) Harmonic field amplitude (top) and pointwise phase-matching factor (bottom) along the output port in the *y-*direction, with representative positions B, C, and D marked with circles. (b) Amplitude (colormap) and phase (white arrows) distributions of the term contributing to point B. (c) Row-wise vector summation path at point B, where color gradient (blue → red) indicates the sequence of vector addition. The resultant vector (from the origin to the red dot) corresponds to the harmonic field strength of point B in (a). (d, e) Distribution of and interference-based vector summation processes for points C; (f, g) the corresponding results for point D.

**Fig. S6** **Phase distribution of** **in a conventional GMR structure.** Under TE_10_ mode, the LN waveguide thickness = 1400 nm. The phase map suggests constructive interference of the subharmonic contributions along the *y-*direction, but destructive interference along the *x*-direction. This feature ultimately gives rise to the elliptical looping superposition path presented in Fig. 3f.

**Fig. S7.** Column-wise summation of the phasor corresponding to Fig. 3f. Despite the highly irregular phase accumulation pattern, the summation yields the same phase-matching factor and final harmonic field amplitude. The blue and red dots indicate the starting and ending positions of the vector arrows, respectively.

**Fig. S8. Strategy for enhancing phase matching factor via oblique incidence.** (a) Field enhancement factor (left axis, dots) and harmonic generation efficiency (right axis, lines) as functions of incidence angle. At 41.95°, the efficiency is only slightly lower than that under normal incidence. The inset shows the corresponding fundamental field distribution. (b) Spatial distribution of the generalized effective excitation source (proportional to) in a nonlinear waveguide at 41.95°, with the white arrows in the background denoting the phaser distribution of. (c) Vectorial superposition of the phasor, yielding a phase-matching factor of 0.13.  Although oblique incidence breaks the symmetry of the microscopic nonlinear sources, the interference of subharmonic waves still follows multiple characteristic elliptical superposition trajectory provided by the GFIM.

**Fig. S9.** **Quasi-BIC-based structure with improved phase matching.** (a) Transmission spectrum of a quasi-bound state in the continuum (quasi-BIC) structure reported in Reference ^[4]^.  The inset shows the corresponding electric field distribution at the resonance wavelength. (b) Spatial distribution of the microscopic nonlinear sources (characterized by) in the LN waveguide. (c) Vectorial superposition of the phasor. A phase-matching factor of 0.28 is achieved, indicating a moderate improvement when the thickness of nonlinear waveguide core is reduced to less than a single interference period.

**Fig. S10 Thickness tuning of a bilayer waveguide for enhanced nonlinear efficiency.** (a) Dispersion relation as a function of LN thickness (= 400 nm). (b) Variation of the field enhancement factor (left axis, blue line) and SHG efficiency (right axis, red line) with LN thickness. The SHG efficiency reached 2.75% at 800nm-thick lithium niobate. (c) Phase-matching factor corresponding to= 600, 700, 800 nm. (d) Dispersion relation as a function of silicon thickness (= 700 nm). (e) Variation of the field enhancement factor (left axis, blue) and SHG efficiency (right axis, red) with silicon thickness. (f) Phase-matching factor corresponding to= 395, 400, 405 nm. Under these favorable phase-matching conditions, increasing the thickness of either the LN or Si waveguide layer leads to a continuous rise in, which remains on the order of 10^4^, providing a practical approach to achieving high SHG efficiency through thickness tuning. The SHG efficiency for 405nm-thick silicon layer is 6.8×10^-3^.

**Fig. S11. Phase distribution of** **in optimized dual-layer GMR structure under TE_10_ mode pumping.** The slowly varying phase in the regions of strong fundamental field enhancement markedly promotes the increase of the phase-matching factor. Structural parameters:.

**Fig. S12. Conversion efficiency obtained from a coupled simulation model of fundamental and second-harmonic waves.** This simulation is applicable to the depleted-pump regime, as validated by the overall energy conservation. The power of fundamental frequency (FF, red) and second harmonic (SH) from both the transmission (green) and reflection (blue) ports are considered.

**Fig. S13** **Harmonic field distributions within the optimized GMR structure for a PMMA width of 900 nm.** (a) Spatial distribution of the harmonic field at a power density of 2 kW/cm², with the color bar indicating the maximum and minimum values. (b-d) Magnitudes of the harmonic fields at the three corresponding labeled ports. The observed spatial uniformity indicates an approximately plane-wave radiation.

**References**

[1]	L. Huang, R. Jin, C. Zhou, et al., "Ultrahigh-Q guided mode resonances in an All-dielectric metasurface," *Nature Communications* **14**, no. 1 (2023): 3433.

[2]	G. Quaranta, G. Basset, O. J. F. Martin, and B. Gallinet, "Recent Advances in Resonant Waveguide Gratings," *Laser Photonics Rev.* (2018): 1800017.

[3]	Y. Kivshar, "All-dielectric meta-optics and non-linear nanophotonics," *National Science Review* (2018): 15.

[4]	H. Jiang, K. Sun, Y. Jia, et al., "Tunable Second Harmonic Generation with Large Enhancement in A Nonlocal All-Dielectric Metasurface Over A Broad Spectral Range," *Advanced Optical Materials* **12**, no. 17 (2024): 2303229.
